# Changes in smoking and alcohol use following the diagnosis of an oral potentially malignant disorder: a retrospective pilot study

**DOI:** 10.1186/s12903-026-07820-x

**Published:** 2026-02-04

**Authors:** Assia Tsyvian, Ameena Nizar Beema, Aia Naksho, Joey-Bahige Chammas, Jordan Gigliotti, Firoozeh Samim, Nicholas Makhoul, Amal Idrissi Janati

**Affiliations:** 1 Faculty of Dental Medicine and Oral Health Sciences, McGill University, 2001 McGill College Avenue, Suite 500, Montreal, QC H3A 1G1 Canada; 2 Department of Dentistry and Oral and Maxillofacial Surgery, Montreal General Hospital, Montreal, QC Canada

**Keywords:** Oral potentially malignant disorders, Smoking, Alcohol, Smoking cessation, Cancer prevention

## Abstract

**Background:**

Smoking and alcohol consumption are major risk factors for oral potentially malignant disorders (OPMDs), yet the extent to which an OPMD diagnosis prompts behavioral change remains unclear. This study aimed to describe the changes in smoking and alcohol consumption in patients after an OPMD diagnosis and explore the role of demographic and clinical factors in these behavioral changes.

**Methods:**

A retrospective cohort study was conducted in the Department of Oral and Maxillofacial Surgery at the McGill University Health Centre, including OPMD patients from 2018 to 2023. Eligible patients had a confirmed OPMD diagnosis, no prior history of head and neck cancer, and at least six months of follow-up. Those without smoking or alcohol data were excluded. Descriptive statistics, McNemar’s and Wilcoxon signed-rank tests to assess behavioral changes, and logistic regression to identify factors associated with continued smoking and alcohol use were used.

**Results:**

The sample composed of 82 patients, 30 (36.6%) were female, with a mean age at diagnosis of 64 years. Leukoplakia (82.9%) was the most common OPMD, and the tongue was the most affected site (42.7%). Median follow-up was 29 months. Most patients (76.8%) had a histologic diagnosis of either mild or moderate dysplasia. At the time of diagnosis, about one-third were active smokers or moderate-to-heavy drinkers. Smoking prevalence decreased from 30.5% at diagnosis to 22.5% post-diagnosis, although this reduction did not reach statistical significance (*p* = 0.06), while moderate-to-heavy alcohol use remained largely unchanged (31.7% vs. 30.5%, *p* = 1.00). Younger patients were less likely to change smoking habits (OR = 0.96, 95% CI 0.91–1.00; *p* = 0.042). Patients from lower socioeconomic backgrounds had lower odds of alcohol use post-diagnosis (OR = 0.26, 95% CI 0.08–0.82; *p* = 0.021).

**Conclusion:**

The findings suggest that smoking cessation is achievable post-diagnosis, whereas reducing alcohol intake may require more targeted interventions. Resistance to behavior change among younger patients may explain rising oral cancer rates in this population.

## Introduction

Oral premalignant lesions, now referred to as oral potentially malignant disorders (OPMDs) as endorsed by the World Health Organization (WHO) classification on Head and Neck Tumors [1], affect approximately 4.5% of the global population, with a higher prevalence observed among men compared to women [2, 3]. Geographical variation in prevalence is evident, with the highest rates reported in Asian, South American, and Caribbean populations, largely reflecting regional patterns of tobacco and alcohol consumption [4]. These lesions are clinically significant, contributing to 17% to 35% of all new cases of oral cavity cancer [4], with an annual malignant transformation rate ranging from 1.4% to 36% [5]. Given their potential for malignancy, early detection of OPMDs, particularly in high-risk populations, remains crucial to reducing morbidity and mortality [6]. While many OPMDs may remain stable or even regress, the potential for unpredictable malignant transformation remains a significant concern. Consequently, a key challenge for clinicians managing these patients is accurately identifying those lesions at greatest risk of progression [7–9].

Commonly recognized OPMDs include leukoplakia, erythroplakia, oral submucous fibrosis (OSMF), proliferative verrucous leukoplakia, and oral lichen planus [10, 11]. These lesions exhibit distinct clinical and histopathological features, with certain types, such as heterogeneous erythroplakia and erythroleukoplakia with dysplasia, demonstrating the highest rates of malignant transformation [6, 12].

Cigarette smoking, smokeless tobacco use, alcohol consumption, and areca nut/betel quid chewing are well-established risk factors for these lesions [13–15]. The oral cavity, lined by rapidly dividing stratified squamous epithelium, is highly vulnerable to these carcinogenic insults. Chronic exposure induces molecular and microenvironmental alterations, leading to dysplasia or hyperkeratosis [16]. Dysplasia within these lesions signifies a heightened risk of malignant transformation [17]. OPMDs exemplify the “field change” phenomenon [18, 19] where chronic carcinogen exposure causes widespread molecular alterations, elevating cancer risk throughout the oral cavity and upper aerodigestive tract [20].

Given this elevated risk, clinicians routinely advise these patients to quit smoking and reduce alcohol consumption to mitigate further disease progression and the development of new lesions. However, it remains uncertain to what extent receiving an OPMD diagnosis and such recommendations translate into meaningful behavioral changes. Understanding whether patients alter or persist in their tobacco and alcohol use following diagnosis and identifying demographic and clinical factors associated with such behavioral changes are essential to designing effective preventive interventions.

Therefore, this study primarily aimed to examine changes in smoking and alcohol consumption following an OPMD diagnosis and secondarily to investigate demographic and clinical factors linked to continued smoking and heavy alcohol use. Assessing patterns of cessation, reduction, and persistence will help identify barriers and determinants of behavior change, providing insights to support targeted interventions that reduce malignant transformation risk and improve long-term outcomes.

## Methods

We conducted a retrospective cohort study at the Department of Dentistry and Oral and Maxillofacial Surgery at the McGill University Health Centre (MUHC) in Quebec, Canada. Using the department’s electronic medical records, we identified patients diagnosed with an OPMD between January 2016 and December 2023. Patients were eligible for inclusion if they were 18 years or older, had a diagnosis of an OPMD confirmed through histopathological analysis, and had a follow-up period of at least six months. Those with a prior history of head and neck cancer were excluded from the study. Data were collected through comprehensive chart reviews, capturing socio-demographic variables (age, sex, socioeconomic status), clinical characteristics (OPMD site, dysplasia severity, and biopsy type), and key outcomes such as malignant transformation and behavioral factors (smoking and alcohol consumption) at both before diagnosis (baseline) and at the last follow-up visit. Socioeconomic status was assessed using the Material and Social Deprivation Index (MSDI) developed by the Institut national de santé publique du Québec [21], which links patients’ postal codes to dissemination areas categorized into quintiles based on education, employment, and income. For analysis, deprivation status was dichotomized as “deprived” (quintiles 4–5) and “not deprived” (quintiles 1–3).

Smoking cessation counseling in this clinic was provided by attending clinicians during routine visits. Counseling generally followed the principles of the 5 A’s model - Ask, Advise, Assess, Assist, Arrange, an evidence-based framework recommended for brief tobacco interventions in healthcare settings [22]. Patients were advised on the risks of continued smoking, the benefits of cessation, and strategies such as behavioral change, and reduction plans. Pharmacotherapy (e.g., nicotine replacement therapy) [23] was occasionally discussed if clinically appropriate, although prescription data were not consistently documented.

Alcohol moderation advice was also provided by attending clinicians as part of routine OPMD counseling. Discussions were based on brief intervention principles aligned with validated approaches such as brief motivational strategies shown to reduce hazardous alcohol use [24]. Patients were informed about recommended weekly limits [25, 26], the synergistic carcinogenic effect of combined alcohol and tobacco use, and the importance of reducing consumption after an OPMD diagnosis. However, the intensity and duration of these interventions were not standardized in the medical records.

Management of OPMD patients at this center follows standard-of-care norms for these lesions, with clinical decisions guided by lesion characteristics, clinical findings, and clinician judgment. For severe dysplasia, lesions are typically excised immediately. Follow-up visits after excision are generally scheduled every 3 months for the first 2 years, then every 6 months for the next 3 years, after which patients are discharged from the clinic and advised to continue routine dental examinations with their dentist. For mild dysplasia, lesions are typically monitored rather than excised, following the same follow-up schedule of every 3 months for 2 years and every 6 months for next 3 years, although patients may be dismissed sooner if closely monitored by their dentist. At each visit, clinical data such as lesion site, size, appearance, symptomatology, and habit status are documented.

Moderate dysplasia represents a “grey area”, where clinicians may choose excision or surveillance based on additional risk factors, including lesion site, size, patient sex (females are considered at slightly higher risk), and tobacco use history. Decisions may vary between clinicians and between patients, as there are no universally accepted guidelines for this category. Follow-up frequency for moderate dysplasia mirrors the schedules described above, depending on whether excision is performed, or the lesion is observed.

Smoking and alcohol consumption were categorized using the Centers for Disease Control and Prevention (CDC) guidelines. Based on the weekly alcohol intake, participants were classified into four groups: non-drinkers or infrequent drinkers (1–11 drinks per year), light drinkers (up to 3 drinks per week), moderate drinkers (more than 3 but no more than 7 drinks per week for women or 14 for men), and heavy drinkers (more than 7 drinks per week for women and more than 14 for men). Based on smoking history, participants were categorized into three groups: never smokers, former smokers, and current smokers [27]. Former smokers were defined as those who had abstained from smoking for at least one year prior to diagnosis. The duration of smoking cessation among current and former smokers was documented. Tobacco exposure was measured using the “pack-year” metric, which quantifies cumulative exposure, with one pack-year equivalent to smoking one pack (20 cigarettes) per day for one year. The study received ethical approval from the Research Ethics Board of the MUHC Research Institute (2024–10231).

### Statistical analysis

Descriptive statistics were used to summarize patients’ sociodemographic characteristics, clinical diagnoses, site of lesion, degree of dysplasia, type of biopsy received, and smoking and alcohol consumption patterns. Categorical variables were reported as percentages, while continuous variables were presented as means with standard deviations or as medians with interquartile ranges (IQR) for non-normally distributed data. Changes in categorical and continuous variables between diagnosis and the last follow-up were assessed using McNemar’s test and Wilcoxon signed-rank test, respectively. To identify factors associated with active smoking and moderate-to-heavy alcohol consumption post-diagnosis, a binary logistic regression analyses were performed. Independent variables included: age at diagnosis, sex, socioeconomic status (collapsed into two categories: deprived vs. not deprived), degree of dysplasia, biopsy type (incisional vs. excisional), and alcohol or smoking status pre- and post-diagnosis. Odds ratios (ORs) and 95% confidence intervals (CIs) were calculated to assess the associations between various independent variables and being a current smoker or moderate-to-heavy alcohol drinker post-diagnosis. For each outcome, only univariate analyses were conducted as the number of patients with the outcome was small.

## Results

A total of 144 patients were initially identified, of whom 82 met the inclusion criteria. Among the included patients, the mean age at diagnosis was 64.12 years (range: 33–97). The cohort consisted of 52 males (63.4%) and 30 females (36.6%). Based on the social deprivation index, 29 participants (35.5%) were classified as deprived, while 43 (52.4%) were not deprived. Data on deprivation index was unavailable for 10 participants (12.2%). The most common clinical diagnosis was leukoplakia, observed in 68 patients (82.9%). Among these, homogeneous leukoplakia accounted for 44 cases (53.7%), while non-homogeneous leukoplakia was identified in 24 cases (29.3%). A breakdown of leukoplakia subtypes and other OPMDs is provided in Table 1. In our cohort, the distribution of dysplasia severity was as follows: mild dysplasia in 31 (37.8%) cases, moderate dysplasia in 32 (39.0%), and severe dysplasia in 17 (20.7%) cases. The three most frequent sites of lesions were the tongue (42.7%), buccal mucosa (17.1%), and gingiva (13.4%). Excisional biopsies were performed in 53.7% of cases, while 46.3% underwent incisional biopsy. During the follow-up period (mean: 29.4. months, range: 6.8–93.3 months), 18.3% of patients experienced disease progression, including recurrence, clinical change, or malignant transformation.

**Table 1:** Sociodemographic characteristics, clinical diagnosis, treatments, and behavioral risk factors in oral potentially malignant disorders study participants

Characteristics	Overall, *n* = 82
Age at surgery, in years: Mean (SD)	64.1 (12.3)
Gender, n (%)	
Male	52 (63.4)
Female	30 (36.6)
Deprivation index, n (%)	
Deprived	29 (35.5)
Not deprived	43 (52.4)
Unknown	10 (11.4)
Clinical diagnosis, n (%)	
Leukoplakia	68 (82.9)
Homogenous leukoplakia	44 (53.7)
Non-homogenous leukoplakia	24 (29.3)
Erythroplakia	3 (3.7)
Oral lichen planus	2 (2.4)
Actinic cheilitis	5 (6.1)
Focal dysplasia	2 (2.4)
Mucosal fibroma with epithelial dysplasia	1 (1.2)
Moderate dysplasia	1 (1.2)
Site, n (%)	
Tongue	35 (42.7)
Buccal Mucosa	14 (17.1)
Gingiva	11 (13.4)
Lip	7 (8.5)
Floor of mouth	5 (6.1)
Palate	4 (4.9)
Others	6 (7.3)
Dysplasia degree n (%)	
Mild	31 (37.8)
Moderate	32 (39.0)
Severe	17 (20.7)
Biopsy type received, n (%)	
Excisional	44 (53.7)
Incisional	38 (46.3)
Progression (recurrence, clinical change or malignant transformation), n (%)	
Yes	15 (18.3)
No	67 (81.7)
Duration of follow-up in months: Median (IQR); [min-max]	29.4 (34.5); [7–93]

*IQR* Interquartile Range

### Smoking patterns and associated factors

At diagnosis, 25 participants (30.5%) were current cigarette smokers, 26 (31.7%) were former smokers, and 31 (37.8%) had never smoked. By the last follow-up visit, 18 (22.0%) continued to smoke, 31 (37.8%) were former smokers, and 31 (37.8%) remained never smokers. Although the proportion of current smokers decreased after diagnosis (30.5% at diagnosis vs. 22.0% post-treatment), this change did not reach statistical significance according to McNemar’s test (*p* = 0.063). Among those who continued smoking, the intensity of cigarette use declined significantly, with the median number of cigarettes per day decreasing from 13 to 10 (Wilcoxon signed-rank test, *p* = 0.042) (Table 2).

**Table 2:** Changes in cigarette smoking and alcohol drinking in OPMD study cohort

Characteristics	Pre-diagnosis	Post-diagnosis	*P* value^†^
Active cigarette smokers, n (%)			
Yes	25 (30.5)	18 (22.0)	0.063
No (never or former)	57 (69.5)	62 (75.6)	
Missing		2 (2.4)	
Number of cigarettes smoked per day for participants who did not quit smoking, median (IQR)	13 (11.5)	10 (13.8)	0.042
Substance smoked at diagnosis (among current smokers)			
Cigarette	20 (80)	14 (77.8)	0.317
Cannabis	2 (8)	2 (11.1)	
Cigarette + Cannabis/cigar/betel	3 (12)	2 (11.1)	
Alcohol drinking, n (%)			
Yes	49(59.8)	45 (54.9)	0.453
No (Never or former)	33 (40.2)	36 (43.9)	
Missing	-	1 (1.2)	
Moderate-heavy alcohol drinking^††^, n (%)			
Yes	26 (31.7)	25 (30.5)	1.00
No*	56 (68.3)	57 (69.5)	

^†^: *p* value for comparison test between two related groups (McNemar’s test for categorical variables and Wilcoxon signed-rank test for continuous variables); ^††^: Moderate drinker = more than 3/week and ≤ 7/week for women and ≤ 14/week for men; Heavy drinker = more than 7/week for women and > 14/week for men; *: being either non-, infrequent or light drinker

To identify potential predictors of continued smoking post-diagnosis, univariate logistic regression analyses were performed for relevant clinical and sociodemographic variables (Table 3). Age at diagnosis was borderline associated with smoking status after diagnosis, with increasing age associated with lower odds of being a current smoker (OR = 0.96, 95% CI: 0.91–1.00; *p* = 0.042). None of the other variables, including gender, socioeconomic status, severity of dysplasia, biopsy type, or alcohol consumption (at diagnosis or follow-up), showed a significant association with smoking behavior after diagnosis (Table 3).

**Table 3:** Regression analysis to identify factors associated with active cigarette smoker after OPMD diagnosis

Independent variables	OR (95% CI); *p* value
Age at surgery (in years)	0.96 (0.91–1.00); *p* = 0.042
Gender (0 = Male; Female = 1)	1.46 (0.50–4.22); *p* = 0.491
Social deprivation Index (0 = Not deprived; 1 = Deprived)	0.66 (0.20–2.17); *p* = 0.495
Biopsy (0 = Incisional; Excisional = 1)	1.57 (0.54–4.58); *p* = 0.408
Dysplasia degree	
Moderate (reference, mild)	1.89 (0.59–6.06); *p* = 0.282
Severe (reference, mild)	0.64 (0.11–3.63); *p* = 0.615
Moderate-heavy alcohol drinking pre-diagnosis (0 = No; 1 = Yes)	0.87 (0.03–2.79); *p* = 0.815
Moderate-heavy alcohol drinking post-diagnosis (0 = No; 1 = Yes)	1.56 (0.52–4.65); *p =* 0.429

*OR* Odds ratio

### Alcohol consumption patterns and associated factors

Alcohol consumption patterns showed minimal variation over time. At diagnosis, 56 participants (68.3%) were infrequent, or light drinkers, while 26 (31.7%) were moderate-to-heavy drinkers. At follow-up, these proportions remained largely stable, with 57 participants (69.5%) in the infrequent/light drinking group and 25 (30.5%) in the moderate-to-heavy drinking group (see Table 2). McNemar’s test did not indicate a statistically significant difference in the proportion of moderate-to-heavy alcohol drinkers between diagnosis and follow-up (*p* = 1.00), suggesting that alcohol use remained relatively unchanged.

Upon univariate logistic regression analyses to identify factors associated with moderate-to-heavy alcohol consumption post-diagnosis (Table 4), socioeconomic status was significantly associated, with individuals from deprived areas having lower odds of alcohol use post-diagnosis (OR = 0.26, 95% CI: 0.08–0.82; *p* = 0.021). Moderate dysplasia showed a borderline association with lower odds of post-diagnosis alcohol use (OR = 0.32, 95% CI: 0.10–1.00; *p* = 0.050). All other variables, including age, sex, type of surgery, severe degree of dysplasia, and smoking status (at diagnosis or follow-up), were not significantly associated with alcohol consumption (Table 4).

**Table 4:** Regression analysis to identify factors associated with being a moderate-to-heavy alcohol drinker after OPMD diagnosis

Independent variables	OR (95% CI); *p* value
Age at surgery (in years)	0.97 (0.93–1.01); *p* = 0.085
Gender (0 = Male; Female = 1)	0.58 (0.21–1.6); *p* = 0.288
Social deprivation Index (0 = Not deprived; 1 = Deprived)	0.26 (0.08–0.82); *p* = 0.021
Biopsy (0 = Incisional; Excisional = 1)	0.72 (0.28–1.85); *p* = 0.49
Dysplasia degree	
Moderate^†^ (reference, mild)	0.32 (0.10–1.00); *p* = 0.050
Severe ^††^ (reference, mild)	0.58 (0.16–2.04); *p* = 0.394
Active cigarette smoking pre-diagnosis (0 = No; 1 = Yes)	1.07 (0.4–3.05); *p* = 0.844
Active cigarette smoking post-diagnosis (0 = No; 1 = Yes)	1.52 (0.52–4.65); *p* = 0.429

*OR* Odds ratio

## Discussion

Smoking is a well-established risk factor for the development and progression of OPMDs. Current smokers have significantly higher odds of developing OPMDs compared to never smokers. For instance, a case-control study in Puerto Rico found that current smoking was associated with a more than fourfold increase in the risk of OPMDs, with an adjusted odds ratio (OR) of 4.32 (95% CI: 1.99–9.38), while former smokers had a considerably reduced risk (OR = 1.47; 95% CI: 0.67–3.21) compared to never smokers [28].

Importantly, smoking cessation has been shown to reduce the risk of malignant transformation of OPMDs. According to one study, cessation of smoking and betel nut chewing was associated with a 36% and 62% reduction in the incidence of OPMDs, respectively, and a 26% decrease in malignant transformation [29]. Additionally, the International Agency for Research on Cancer (IARC) has concluded OPMD risk, particularly for leukoplakia, declines following cessation [30]. In our cohort, a decrease in current smokers alongside an increase in former smokers over time suggests a positive behavioral shift post-diagnosis. This change is clinically significant, as tobacco cessation may lead to regression or stabilization of dysplastic lesions and reduce the likelihood of malignant transformation [31].

Furthermore, the diagnosis of an OPMD itself may serve as a catalyst for smoking cessation. Studies have shown that a medical diagnosis can influence patients’ decisions to quit smoking, especially when the disease is perceived as severe or life-threatening. Research indicates that patients diagnosed with serious conditions like myocardial infarction or lung cancer exhibit higher smoking cessation rates compared to those with less severe diagnoses. Specifically, a study by Taniguchi et al. found that the smoking cessation rate after diagnosis in patients with acute myocardial infarction and lung cancer exceeded 59%, whereas it ranged between 31 and 40% in patients with colorectal and stomach cancer [32]. In our study, despite the absence of a structured tobacco or alcohol cessation program, a significant reduction in smoking was observed following OPMD diagnosis. This suggests that the awareness of having a potentially malignant disorder may motivate individuals to adopt healthier behaviors, including cessation of smoking. Continued alcohol use, especially at moderate to heavy levels, has been associated with poorer outcomes and an elevated risk of malignant transformation [33]. Despite this, previous studies have also reported a similar inertia in alcohol behavior post-diagnosis [34], attributing it to the underemphasis of alcohol risks during counseling [35] or limited awareness among patients regarding its role in oral carcinogenesis [36]. Although our study assessed alcohol consumption as a binary variable (use vs. cessation), emerging evidence suggests that the type of alcoholic beverage may influence oral mucosal changes differently. Beverages with higher ethanol concentration, such as spirits, deliver greater mucosal exposure to acetaldehyde, a Group I carcinogen, which can potentiate epithelial dysplasia and malignant transformation more strongly than beverages with lower ethanol content (e.g., beer or wine) [37]. Additionally, fermented beverages may contain yeast-derived compounds capable of locally increasing acetaldehyde levels, further amplifying risk [38]. Because our retrospective dataset did not capture the specific type, quantity, or pattern of alcohol use, we were unable to explore these distinctions, representing an important area for future research.

### Post-diagnosis smoking and alcohol use: key predictors

Age at diagnosis was the only variable significantly associated with smoking post-diagnosis. Older individuals were less likely to continue smoking, suggesting that increasing age may serve as a protective factor. This is consistent with existing literature indicating that older adults often demonstrate greater health consciousness and risk aversion following serious diagnoses. Boyle et al. found that higher risk aversion in older individuals was associated with more cautious decision-making, which may explain the reduction in smoking behavior [39].

In the case of alcohol consumption, both age at diagnosis and deprivation index were significantly associated with post-diagnosis behavior. Older individuals had lower odds of engaging in moderate-to-heavy alcohol use, which may reflect increased health awareness and lifestyle adjustments that often accompany aging. As reported by Molander et al., alcohol consumption tends to decline from midlife into older adulthood, driven by health concerns, medication interactions, and shifting personal priorities [40]. Participants from more deprived backgrounds were also less likely to consume alcohol. This finding may be explained by socioeconomic and cultural factors that influence health behaviors; as noted in a systematic review by Bryden et al., alcohol use can be shaped by community-level deprivation, with lower consumption in some deprived populations potentially due to reduced affordability, availability, or differing social norms [41].

All other variables, including sex, biopsy type (excisional vs. incisional), and dysplasia severity, were not significantly associated with post-diagnosis smoking or alcohol consumption. Neither moderate nor severe dysplasia appeared to influence patients’ decisions to continue these behaviors, suggesting that histological disease severity may not be adequately understood or perceived as a trigger for behavioral change.

### Interaction between smoking and alcohol use

The synergistic interaction between tobacco and alcohol is well established in oral carcinogenesis, with combined exposure conferring a markedly higher risk than either habit alone [42]. Alcohol increases mucosal permeability to tobacco carcinogens and enhances local acetaldehyde production, thereby amplifying mutagenic effects [43]. Notably, epidemiological evidence indicates that cessation of at least one of these behaviors can substantially reduce oral cancer risk, even when the other exposure persists. Marron et al., using pooled INHANCE data, demonstrated that smoking cessation lowered head and neck cancer risk across all anatomical subsites, irrespective of alcohol use status [44]. In our cohort, smoking prevalence declined significantly following diagnosis, whereas alcohol use remained unchanged, suggesting that partial risk reduction through modification of a single high-risk behavior may be a more immediately achievable goal for many patients. From a clinical standpoint, these findings support a pragmatic harm-reduction approach, whereby clinicians actively encourage cessation of at least one habit while promoting control of the other when simultaneous cessation of both tobacco and alcohol is not feasible [45]. Interestingly, population-based evidence indicates that cessation of one behavior can facilitate cessation of the other: in China, stopping alcohol was associated with higher smoking cessation rates [46], while Dawson et al. reported that individuals who quit smoking were roughly three times more likely to stop drinking during follow-up [47]. Together, these studies support the potential benefits of a stepwise harm-reduction strategy that focuses on modifying one habit at a time among co-users.

Despite this well-documented biological interaction, our analysis did not identify a significant cross-behavioral influence, such that alcohol consumption did not predict smoking status post-diagnosis, nor did smoking status influence alcohol use. This finding is consistent with previous study by Lorona et al., where no significant associations were found between alcohol consumption or smoking history at the time of colorectal cancer diagnosis and its subsequent recurrence or mortality [48]. Similarly, tabuchi et al. found that heavy drinking and heavy smoking were independent additive risk factors for subsequent primary cancer [49]. Together, these findings suggest that while biological synergy is well established, behavioral modification of one habit does not necessarily translate into change in the other.

### Clinical implications and evidence-based recommendations

The findings of this study provide several clinically relevant insights into behavioral patterns among patients diagnosed with OPMDs. Although smoking prevalence significantly decreased after diagnosis, alcohol consumption remained unchanged, highlighting a persistent gap in patient perception of alcohol-related risk. Younger patients were less likely to modify either behavior, suggesting that risk communication strategies may need to be tailored by age group. Additionally, patients from socioeconomically deprived backgrounds demonstrated lower odds of post-diagnosis alcohol use, underscoring the influence of social determinants on health behaviors. These results reinforce the importance of targeted counseling at the time of diagnosis and suggest that clinicians may need to deliver clearer, more structured guidance regarding alcohol-related risks, particularly for younger adults.

Evidence-based guidance for the clinical management of OPMDs further reinforces the importance of structured follow-up and risk reduction strategies. Recent consensus statements and guidelines recommend that optimal care for patients with OPMDs should include a thorough examination of the entire oral mucosa, careful documentation of lesion site, size, and clinical subtype, and timely biopsy or re-biopsy of any suspicious, symptomatic, or changing lesions [11, 50, 51]. Risk-stratified recall intervals are advised, with low-risk lesions typically reviewed every 6–12 months and higher-risk lesions (e.g., non-homogeneous leukoplakia, lesions with epithelial dysplasia, or multifocal disease) seen at 3–6-month intervals, particularly in the first few years after diagnosis [11, 46, 48]. Behavioral counseling remains central to management; cessation of tobacco, alcohol, and betel quid use is strongly recommended to reduce malignant transformation risk, with clinicians encouraged to provide brief interventions and, where possible, refer patients to structured cessation services [50–52]. Multidisciplinary assessment linking primary care, oral medicine, oral and maxillofacial surgery, pathology, and oncology is recommended for high-risk lesions, progressive dysplasia, or recurrent disease to ensure coordinated decision-making and timely intervention [50, 51].

Evidence from a study by Poate et al., conducted in a dedicated oral dysplasia clinic, demonstrated substantial reductions in smoking prevalence following clinician-led cessation counseling, underscoring the effectiveness of formalized intervention frameworks [53]. In addition, outcome-based studies in patients with OPMDs have shown that smoking cessation is associated with improved prognosis; Vladimirov and Schiødt reported a significantly reduced risk of unfavorable events following treatment of OPMDs among patients who quit smoking [29]. Together, these findings strongly suggest that establishing structured tobacco and alcohol cessation programs, along with systematic referral to specialized cessation services, could further enhance the magnitude and sustainability of behavior change and improve clinical outcomes in patients with OPMDs.

This study relies on self-reported data for smoking and alcohol use, which may be subject to recall or social desirability bias. While the design allows observation of behavioral trends over time, residual confounding from unmeasured variables such as psychosocial support, access to cessation resources, or comorbidities cannot be ruled out. Importantly, no biochemical validation (e.g., cotinine testing or carbon monoxide monitoring) [54] was performed to objectively confirm smoking cessation. Similarly, alcohol cessation was assessed solely through self-report, without using indicators such as phosphatidylethanol (PEth) [55], carbohydrate-deficient transferrin (CDT) [56], or ethyl glucuronide (EtG) [57]. The absence of these objective measures may have led to misreporting, potentially overestimating the true rates of cessation in this cohort. or underestimation of continued use. Electronic cigarette (e-cigarette) use was also not assessed in this study, although its relevance to OPMDs and epithelial dysplasia is increasingly recognized. While e-cigarettes contain fewer carcinogens than combustible tobacco, they still expose the oral mucosa to nicotine, formaldehyde, acrolein, and reactive oxygen species, which may contribute to oxidative stress and epithelial injury [58]. Emerging evidence shows that e-cigarette users demonstrate cytological alterations, DNA damage, and dysplastic-like cellular changes, raising concern that vaping may influence OPMD behavior or progression [59]. The lack of e-cigarette data in our cohort limits our ability to evaluate these potential effects and highlights the need for future studies to incorporate this exposure. Additionally, the sample size of 82 patients reflects the natural referral pattern to an Oral and Maxillofacial Surgery clinic, which provides a broad range of services including dentoalveolar surgery, orthognathic surgery, craniomaxillofacial trauma care, oral cancer management, and complex reconstruction, rather than functioning as an OPMD-dedicated service. While this limits broad generalizability, the cohort remains representative of typical OPMD presentations in multidisciplinary clinical settings. Finally, although behavioral change was observed post-diagnosis, the long-term sustainability of these changes and their impact on clinical outcomes remain uncertain.

## Conclusion

The findings of the study, especially the **l**ack of strong behavioral shifts following diagnosis, highlight the limited impact of clinical encounters alone in motivating patients to modify substance use. Despite the known etiological role of tobacco and alcohol in oral carcinogenesis, the observed resistance in behavior changes points toward a need for more structured and intensive cessation support integrated into the care pathway. Early counseling, longitudinal follow-up, and possibly behavioral therapy or pharmacological interventions may be warranted to promote meaningful and sustained changes in these high-risk habits.

## Data Availability

The datasets used and/or analysed during the current study are available from the corresponding author on reasonable request.

## Abbreviations

CDC: Centers for Disease Control and Prevention
CI: Confidence Interval
IQR: Interquartile Range
OPMD: Oral Potentially Malignant Disorders
OR: Odds Ratio
